# Public attitudes towards automated external defibrillators: results of a survey in the Australian general population

**DOI:** 10.3389/fcvm.2023.1178148

**Published:** 2023-06-02

**Authors:** Joshua G. Kovoor, Simone Marschner, Anjalee Amarasekera, Meera Nageswaran, Gregory J. Page, Clara K. Chow, Aravinda Thiagalingam, Pramesh Kovoor

**Affiliations:** ^1^ The Queen Elizabeth Hospital, University of Adelaide, Adelaide, SA, Australia; ^2^ Heart of the Nation, Sydney, NSW, Australia; ^3^ Health and Information, Adelaide, SA, Australia; ^4^ Westmead Applied Research Centre, Faculty of Medicine and Health, University of Sydney, Sydney, NSW, Australia

**Keywords:** automated external defibrillator (AED), public attitude, sudden cardiac arrest, cardiopulmonary resuscitation, society, Australia

## Abstract

**Background:**

Swift defibrillation by lay responders using automated external defibrillators (AEDs) increases survival in out-of-hospital cardiac arrest (OHCA). This study evaluated newly designed yellow–red vs. commonly used green–white signage for AEDs and cabinets and assessed public attitudes to using AEDs during OHCA.

**Methods:**

New yellow–red signage was designed to enable easy identification of AEDs and cabinets. A prospective, cross-sectional study of the Australian public was conducted using an electronic, anonymised questionnaire between November 2021 and June 2022. The validated net promoter score investigated public engagement with the signage. Likert scales and binary comparisons evaluated preference, comfort and likelihood of using AEDs for OHCA.

**Results:**

The yellow–red signage for AED and cabinet was preferred by 73.0% and 88%, respectively, over the green–white counterparts. Only 32% were uncomfortable with using AEDs, and only 19% indicated a low likelihood of using AEDs in OHCA.

**Conclusion:**

The majority of the Australian public surveyed preferred yellow–red over green–white signage for AED and cabinet and indicated comfort and likelihood of using AEDs in OHCA. Steps are necessary to standardise yellow–red signage of AED and cabinet and enable widespread availability of AEDs for public access defibrillation.

## Introduction

Out-of-hospital cardiac arrest (OHCA) is a prevalent global health concern where over nine in 10 patients do not survive, and most die before reaching a hospital (1–4). Rapid defibrillation is crucial to potential survival and long-term quality of life (5, 6). In cases of OHCA, chances of survival decrease by 3% every minute that defibrillation is delayed after cardiopulmonary resuscitation (CPR) is commenced (7). When OHCA occurs in the community, lay responders play a crucial role in giving patients a chance of survival, through alerting emergency medical services (EMS) and initiating CPR and early defibrillation (8). Initial defibrillation by lay first responders is associated with greater OHCA survival than initial defibrillation by dispatched EMS (9). The primary method by which lay responders can deliver rapid defibrillation to OHCA patients is via the use of publicly accessible automated external defibrillators (AEDs), which is safe and effective for improving survival even with no training (10). However, within Australia, despite investments by many governments to increase the number of publicly accessible AEDs found within communities, many OHCA cases still occur over 100 m away from the locations where these are situated, indicating that current coverage is inadequate (11). This paucity of publicly accessible AEDs within communities (12) provides a potential explanation for bystander use of AEDs occurring in under 2% of non-EMS witnessed OHCA cases in Australia (4).

In situations of community OHCA, rapid defibrillation by lay responders relies on AEDs being swiftly identifiable and publicly accessible. The primary method of identification is via signage and the exterior of the cabinet in which the AED is placed. In 2008, the International Liaison Committee on Resuscitation (ILCOR) proposed a sign indicating the presence of AEDs worldwide, utilising a green–white colour combination (13, 14). Further investigations of variants of AED sign designs have also utilised this green–white colour scheme (15). However, it has been demonstrated that public recognition and understanding of current green–white AED signage is limited and no single sign is unanimously recommended by national resuscitation councils or implemented in a standardised fashion in communities worldwide (16).

Colour perception is an important factor influencing human interaction with different environments (17). Past literature has found the colour green to be associated with lower alertness and greater calmness, whereas more vivid colours such as red and yellow have been associated with increased alertness and memory retention (18). Accordingly, the combination of vivid colours such as yellow and red is integral to the marketing strategy of some of the world's top corporations (19). In the emergency of OHCA, signage incorporating primarily vivid colours may be effective in facilitating a lay responder's rapid identification of a publicly accessible AED's location and potentially heightened awareness of their locations generally. Accordingly, we conducted a national survey of the Australian general public to evaluate a proposed new yellow–red sign and cabinet vs. the most commonly used green–white version for identifying AEDs.

## Methods

### Study design and oversight

**Figure 1:**
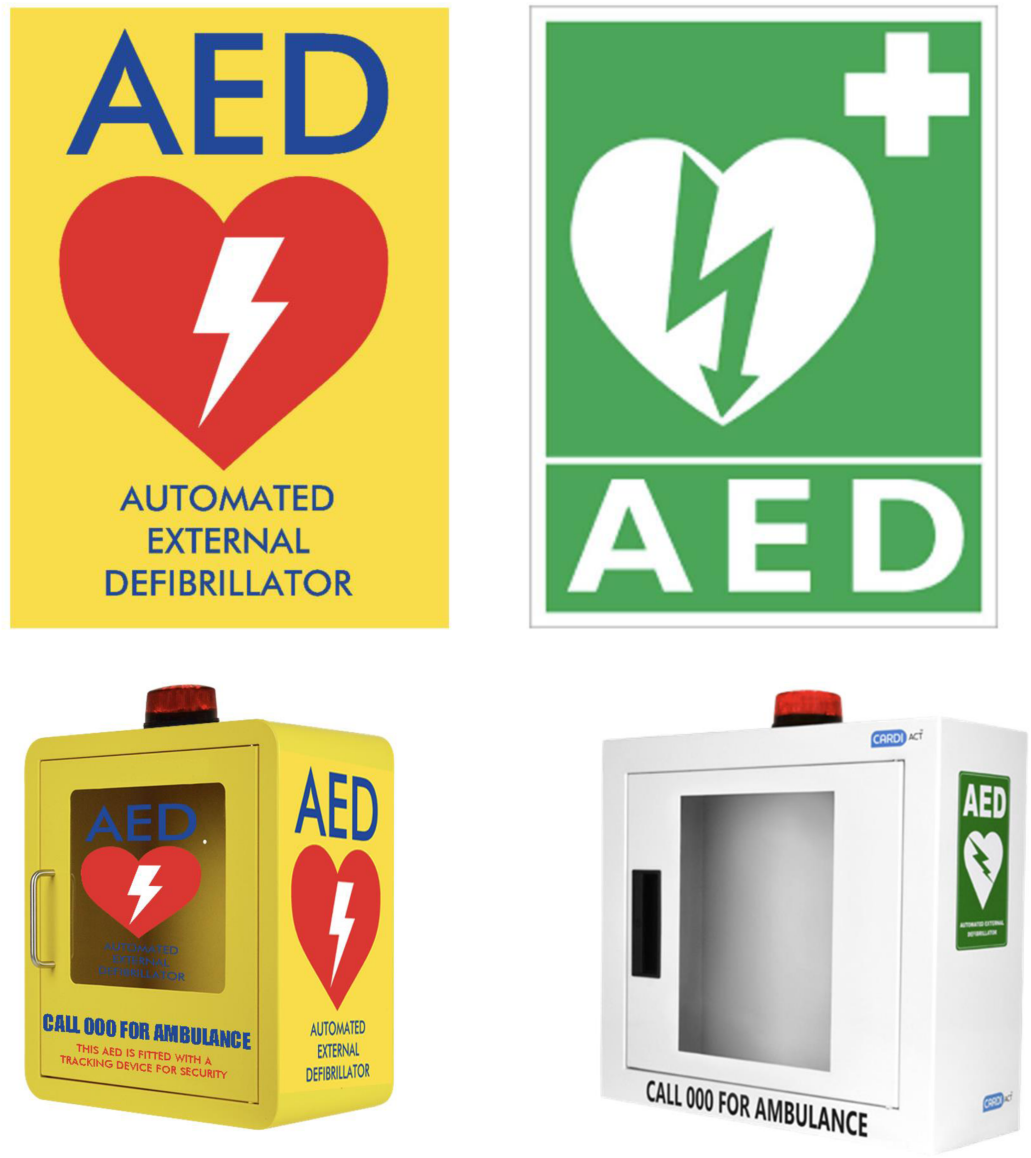
AED signs and cabinets investigated in this study. AED, automated external defibrillator.

This prospective, cross-sectional study was undertaken in collaboration with Heart of the Nation (an initiative of the registered Australian Charity, Our National Heart Pty Limited) and the Westmead Applied Research Centre. It followed the STROBE guidelines for reporting observational studies (20) and was conducted between November 2021 and June 2022 across Australia. The AED signs and cabinets investigated in this study are presented in Figure 1. The evaluated yellow–red sign and cabinet were designed by Heart of the Nation, in accordance with the International Organization for Standardization for Graphical Symbols and Test Methods (ISO 9186-1) (21). As co-authors and collaborators, members of Heart of the Nation and Westmead Applied Research Centre were responsible for the study’s design and execution, including data acquisition, analysis and interpretation. They also critically revised the article for crucial intellectual content and made the final decision to submit the manuscript for publication. Ethical approval was obtained from the Western Sydney Local Health District Human Research Ethics Committee (reference number: 2021/ETH12008).

### Participants and data collection

The study population comprised members of the Australian general population. To ensure that the sample was representative, we included all demographic subgroups, and no restrictions or exclusion criteria were applied. An electronic, anonymised questionnaire was developed using a web application (REDCap, Vanderbilt University, TN, United States) (22) and distributed using emails and social media posts containing the survey link, inviting members of the general public to participate. No random assignment or balancing was conducted.

### Outcome measures

The primary outcome was the validated net promoter score (NPS), which was used to investigate public engagement with the signs and cabinets presented in the survey and provide respective ratios of promoters to detractors (23). Other measures included Likert scales and binary comparisons evaluating the yellow–red vs. green–white signs and cabinets for preference, ease of identification in an emergency such as cardiac arrest and comfort and likelihood of using AEDs in situations of OHCA.

### Statistical analysis

Quantitative data were analysed using descriptive statistics. The proportion of the community that would find the new sign easier to identify in an emergency such as a cardiac arrest, and similarly for the cabinet, was estimated with a 95% confidence interval. Logistic regression models were used to assess the effect of age, ethnicity and region on this proportion. NPS estimates were calculated, and 95% confidence intervals were presented for the new and original sign and cabinet. Ordinal regression models were used to assess the effect of age, ethnicity and region on the NPS for each cabinet and sign. The McNemar–Bowker Test was used to compare the distribution of promoters, passives and detractors between the new and original signs and similarly for the cabinets. Analyses were performed using R (version 4.0.2) (24) packages Gmisc (25) for plot and table output and knitr (26) for reproducible research. *p*-values of less than 0.05 were considered statistically significant unless stated otherwise. The full survey and statistical report can be found in the Supplementary Appendix. Details on the measurement of outcomes, including survey scores and raw data, are available on reasonable request to the corresponding author.

## Results

### Study sample

A total of 2,538 members of the Australian general population participated in the study by clicking on the survey link distributed by email and social media. The data regarding the number of people who had access to the survey link, but did not participate, were not available. The mean age was 30.9 (SD: 14.9) years. Regarding gender, 1,454 (59.4%) were female, 897 (36.6%) male and 70 (2.9%) non-binary, and the remainder preferred not to say. Regarding race and ethnicity, 2,055 (81.0%) were white, 293 (11.5%) Asian, 86 (3.4%) Aboriginal or Torres Strait Islander (ATSI), 36 (1.4%) Pacific Islander, 34 (1.3%) Hispanic, 24 (0.9%) African-American and 7 (0.3%) American Indian. Of the study population, 510 (21.0%) were healthcare workers.

### Preference and ease of identification

**Figure 2:**
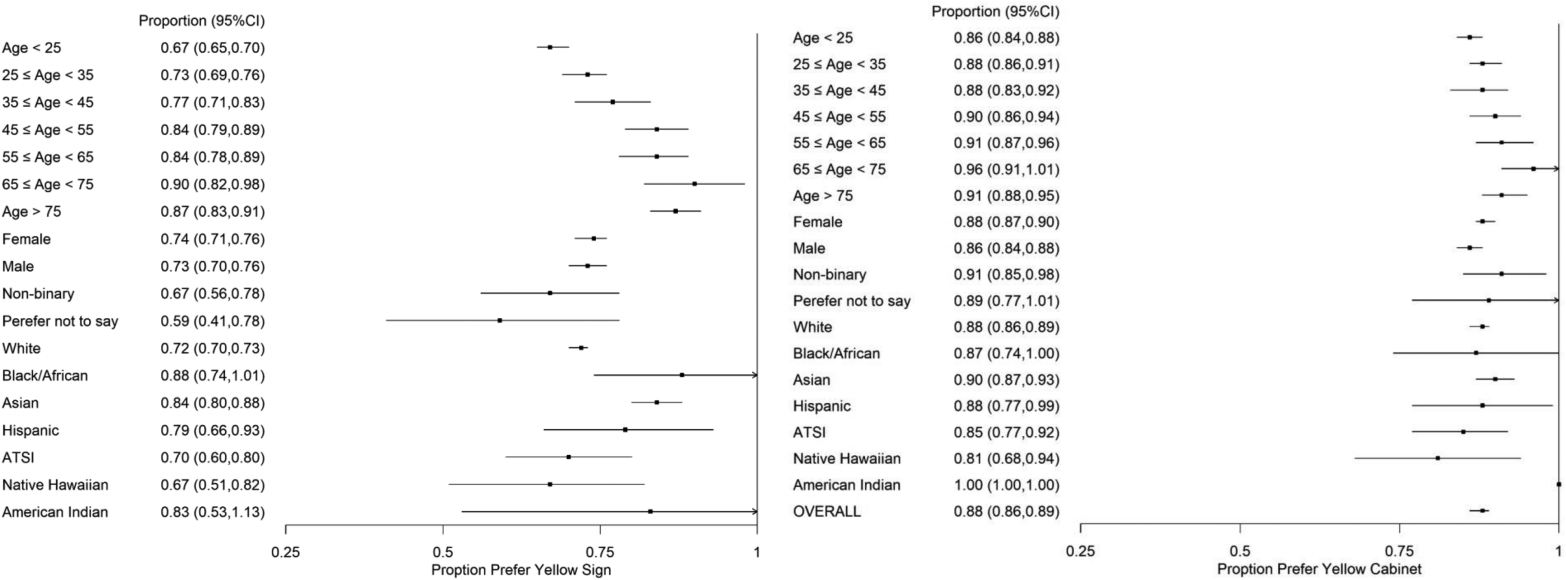
Demographics of yellow–red AED sign and cabinet preference.

The yellow–red sign was preferred by 1,778 (73.0%) as easier to identify in emergencies such as cardiac arrest vs. 658 (27.0%) for green–white. The yellow–red cabinet was reported as easier to identify by 2,139 (87.6%) vs. 302 (12.4%) green–white. With similar rates of preference by gender and ethnicity, older people had the greatest preference for yellow–red signs and cabinets (Figure 2). Age and ethnicity were significantly associated with the ease of identifying yellow–red signs or cabinets (Table 1). The likelihood of preferring the yellow–red over the green–white sign grew by 2% for every additional year of age.

**Table 1:** Variables significantly associated with stronger ease for identifying yellow signs and cabinets^a^.

	Odds ratio and 95% confidence interval	*p*-value
Variables found associated with easily identifying the **yellow–red sign compared to the green–white sign**		
Age	1.024 (1.016–1.031)	<0.0001
Ethnicity: Asian	1.86 (1.33–2.60)	0.0003
Variables found associated with easily identifying the **yellow–red cabinet compared to the green–white cabinet**		
Age	1.014 (1.004–1.023)	0.0038
Ethnicity: Asian	1.70 (1.08–2.77)	0.0213
Ethnicity: White	1.89 (1.08–3.33)	0.0269

^a^ Raw data in Supplementary Appendix.

### Comfort and likelihood of using AEDs

Regarding comfort using AEDs in OHCA, 631 (26.0%) were very comfortable, 684 (28.2%) slightly comfortable, 344 (14.2%) neutral, 499 (20.5%) slightly uncomfortable and 271 (11.2%) very uncomfortable. Regarding the likelihood of using AEDs in OHCA, 1,013 (42.0%) were very likely, 536 (22.2%) slightly likely, 415 (17.2%) neutral, 233 (9.7%) slightly unlikely and 217 (9.0%) very unlikely.

### Public engagement

Within NPS results, the yellow–red AED sign and cabinet demonstrated significantly higher proportions of promoters and lower proportions of detractors, vs. green–white (Table 2). The yellow–red sign achieved an NPS of 0.33 (95% CI 0.30–0.36) vs. −0.41 (95% CI −0.44 to −0.38) for green–white. The yellow–red cabinet achieved an NPS of 0.48 (95% CI 0.45–0.51) vs. −0.61 (95% CI −0.64 to −0.58) for green–white.

**Table 2:** Net promoter score results^a^.

	Promoters	Detractors	Passive	NPS and 95% CI
Green–white sign	19.4%	60.1%	20.5%	−0.41 (−0.44 to −0.38)
Yellow–red sign	53.5%	20.6%	26.0%	0.33 (0.30 to 0.36)
Green–white cabinet	11.6%	72.5%	15.8%	−0.61 (−0.64 to −0.58)
Yellow–red cabinet	62.3%	14.3%	23.4%	0.48 (0.45 to 0.51)

^a^ Raw data in Supplementary Appendix.

## Discussion

This prospective, small, non-representative pilot study of the Australian general population found that yellow–red signs and cabinets may be significantly preferred and reported as easier to identify over green–white counterparts for the public identification of AEDs. Age and ethnicity may be associated with the ease of identifying the yellow–red signs and cabinets. Of note, increased age may be associated with an increased preference for the yellow–red sign over the green–white alternative. It was very encouraging that the majority of the general population may be comfortable in using AEDs in a situation of OHCA and the majority may be likely to use an AED if this situation did arise. In comparison with those of green–white alternatives, yellow–red AED signs and cabinets may have higher proportions of promoters and lower proportions of detractors regarding public engagement.

The societal toll of sudden cardiac arrest is large. Australia experiences over 20,000 sudden cardiac arrests each year, which is associated with annual economic losses of AUD 2 billion (USD 1.42 billion) and productivity losses comparable to those from all cancers combined (27). To reduce sudden cardiac death, specifically that associated with OHCA, societal change is necessary (12). It is known that rapid defibrillation is a necessary complement to CPR for preventing mortality in cases of OHCA (5–7), and it is intuitive that the swift use of an AED by a lay responder in this situation (8–10) relies on them being able to quickly identify the AED's location within a community environment. In this emergency scenario, it is also intuitive that the use of signs utilising vivid colour combinations, such as yellow–red, would likely catch the attention of lay responders quicker and more effectively than the use of placid colour combinations, such as green–white (18). Further, the green–white colour combination is commonly used in society to indicate a range of signs, including those demarcating first aid kits and building exits, and, accordingly, it is likely to be less clear in the public’s psyche as a sign specifically indicating the presence of an AED in life-threatening emergencies. However, given that only yellow–red and green–white colour combinations were evaluated in the present study, future research should consider investigating other vivid colour combinations that are different to these green–white colour combinations, such as blue–red.

There would likely be significant societal benefit from a unique, clearly recognisable sign, utilising a vivid yellow–red colour combination, for the broadly standardised identification of publicly accessible AEDs. The present study provides evidence that members of the Australian general population may engage more with, prefer and more easily identify yellow–red AED signs and cabinets compared with current green–white alternatives. As the public recognition of current green–white AED signage is limited and no single sign is implemented broadly (16), we propose that yellow–red signs and cabinets be considered by public health authorities for the standardised identification of publicly accessible AEDs. We also urge public health authorities to acknowledge the public's willingness to use AED and to take urgent steps to enable widespread availability of AEDs for prompt public access defibrillation in cases of OHCA. However, the issue of signage colour applies to all emergencies within society, especially those that involve EMS vehicles. Signage for emergencies should be vibrant wherever possible, to increase alertness and engagement for the situation in the members of the public that view them (17, 18).

However, colour combinations must be distinct for each respective emergency, to not confuse the lay responders.

This study has multiple limitations. Although the survey was open to all members of the Australian general population and no restrictions or exclusion criteria were applied so that the sample would be representative, potential bias may have been incurred as those that responded to the email and social media invitation to participate may have been those with greater engagement with the content evaluated in the present study. Further, data were not available regarding the number of people who had access to the survey link but did not participate. As the outcomes of the study were self-reported, there is the potential for either under or overreporting based on participant characteristics. Although no socioeconomic restrictions were employed within the study's inclusion criteria, only people within Australia were evaluated, and accordingly the translatability of the present findings to other countries is unknown and requires future investigation. The characteristics of the study population may provide a source of bias and may not be completely representative of the general population, particularly given that the mean age was just over 30 years old, about 60% were of female gender, over 80% were of white race and ethnicity and over 20% were healthcare workers. As no questions were proposed regarding participants’ prior experience with OHCAs, it is challenging to infer from the present survey how and whether the colour of publicly accessible AED signs and cabinets may effectively affect the public attitudes to use a publicly accessible AED in the event of OHCA. In addition to this question, future research should also seek to investigate if participants have ever been involved in an OHCA resuscitation, if they found it difficult to locate an AED and, if so, was it due to AED location, sign or cabinet colour or another reason. These data are crucial to completely describing the role of sign and cabinet colours in influencing public attitudes to AED use in OHCA.

## Conclusions

This prospective, small, non-representative pilot study of the Australian general population found that yellow–red signs and cabinets may be significantly preferred and easier to identify over green–white counterparts for the public identification of AEDs. There may also be a reasonable public willingness to use AEDs in OHCAs. As the public recognition of current green–white AED signage is limited and no single sign is implemented broadly, we propose that yellow–red signs and cabinets be considered for the standardised identification of publicly accessible AEDs. Public health authorities should be encouraged by the public's willingness to use AEDs and initiate steps to have widespread availability of AEDs in out-of-hospital cardiac arrest. However, further major and more representative, public consideration and investigation must be conducted.

## Data Availability

The original contributions presented in the study are included in the article/Supplementary Material, further inquiries can be directed to the corresponding author.
