# Supplementary material for: Public attitudes towards automated external defibrillators: results of a survey in the Australian general population
Source: Front Cardiovasc Med. 2023 Jun 2;10:1178148. doi: 10.3389/fcvm.2023.1178148 (PMC10272715; doi:10.3389/fcvm.2023.1178148)
Supplement: Supplementary file 1 [file Datasheet1.docx]

**SUPPLEMENTARY APPENDIX**

**Signage of Automated External Defibrillators (AED) for Global Identification**

**Ethics approval = WSLHD HREC Approval # 2021/ETH12008 (dated 06/01/2022)**

**Background**

Automated external defibrillators (AEDs) are a safe way to save people having cardiac arrests out of hospital. However, finding AEDs quickly relies on the signs and cabinets marking its location being easily seen.

**Purpose**

To understand which signs and cabinets are most effective for finding AEDs during cardiac arrests.

**Details**

Answers are not identifiable and will remain confidential. Your participation in this study is voluntary and you can withdraw at any time. The study has been approved by Western Sydney Local Health District.

**Consent**

By answering the questions of this survey, you are consenting to participate in this research survey.

**Survey Questions**

***What is your gender?***

1. Female
2. Male
3. Non-binary
4. Prefer not to say

***What is your race/ethnicity? (check all that apply)***

1. White
2. Black/African American
3. Asian
4. Hispanic, any race
5. Aboriginal or Torres Strait Islander
6. Native Hawaiian / Other Pacific Islander
7. American Indian / Alaska Native

***What is your age?***

[Numerical entry]

***Are you a healthcare worker trained in delivering basic life support?***

1. Yes
2. No

***Which sign would be easier to identify in an emergency such as a cardiac arrest?***


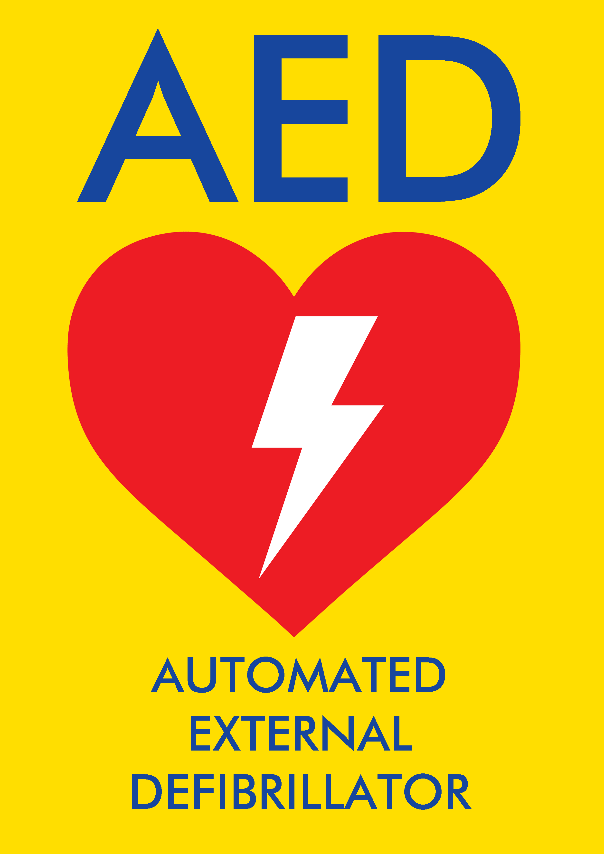

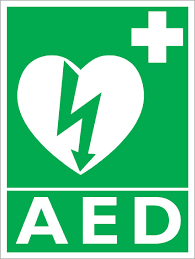


1. Yellow
2. Green

***Which cabinet would be easier to identify in an emergency such as a cardiac arrest?***


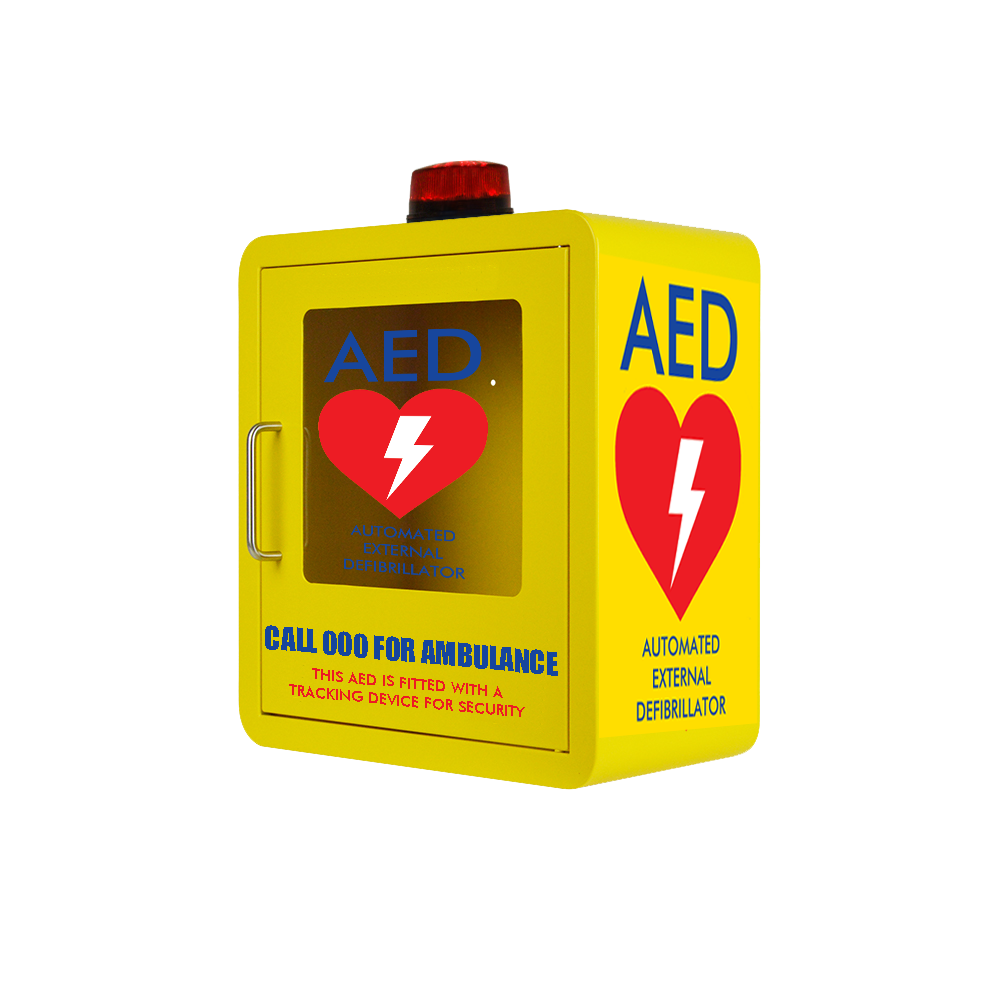

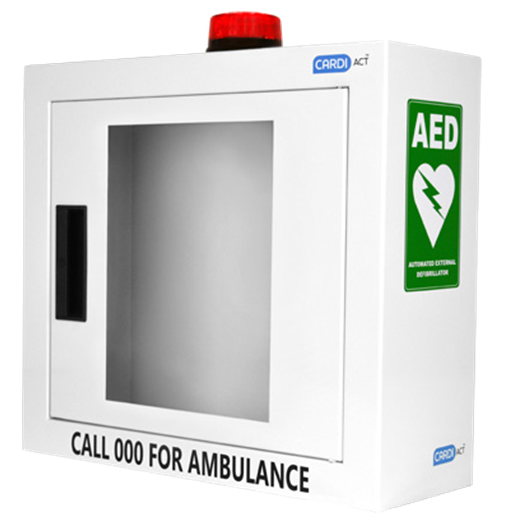


1. Yellow
2. White

***How likely is it that you would recommend this sign for the identification of an automated external defibrillator (AED) in the emergency of a friend or colleague’s cardiac arrest on a scale of 0-10? (0 being least likely, 10 being most likely)***


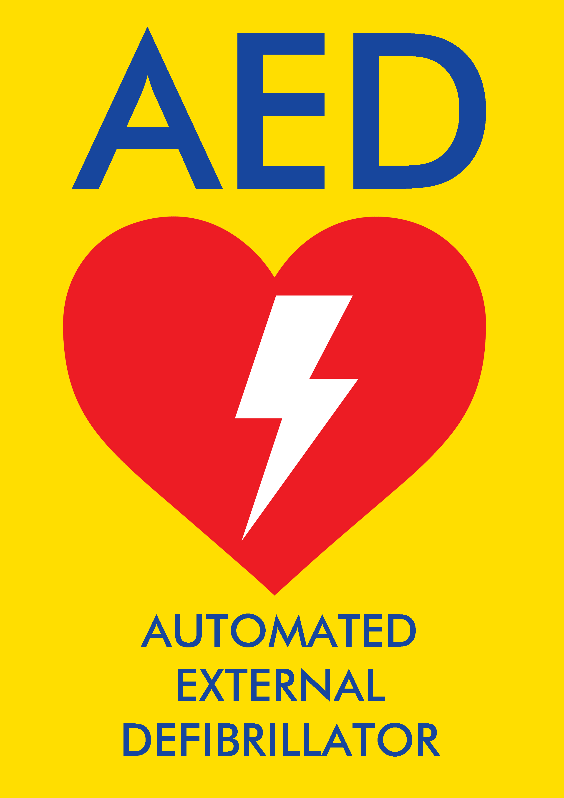


[0-10 numerical scale]

***How likely is it that you would recommend this sign for the identification of an automated external defibrillator (AED) in the emergency of a friend or colleague’s cardiac arrest on a scale of 0-10? (0 being least likely, 10 being most likely)***


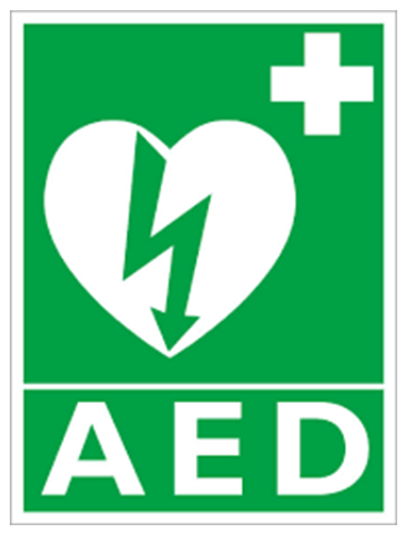


[0-10 numerical scale]

***Based on only on the colour of this cabinet, how likely is it that you would recommend this cabinet for the identification of an automated external defibrillator (AED) in the emergency of a friend or colleague’s cardiac arrest? on a scale of 0-10? (0 being least likely, 10 being most likely)***


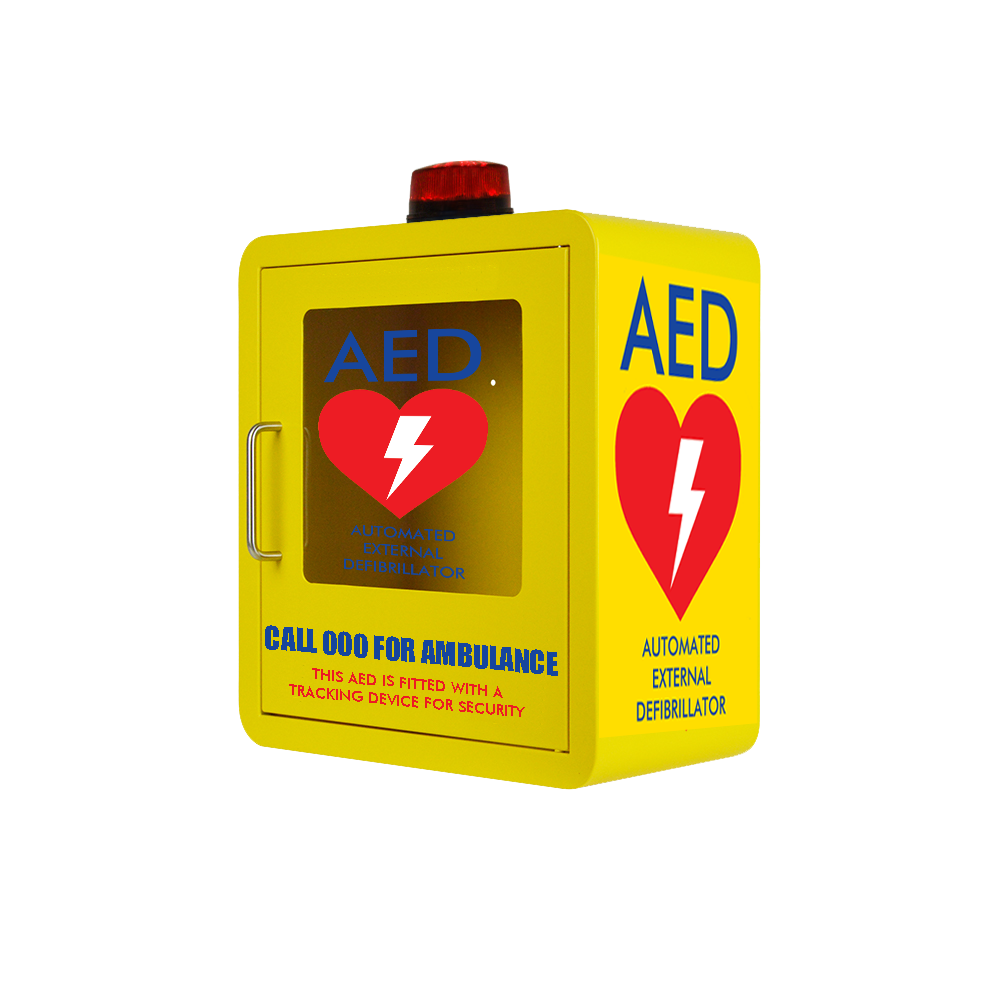


[0-10 numerical scale]

***Based only on the colour of this cabinet, how likely is it that you would recommend this cabinet for the identification of an automated external defibrillator (AED) in the emergency of a friend or colleague’s cardiac arrest on a scale of 0-10? (0 being least likely, 10 being most likely)***


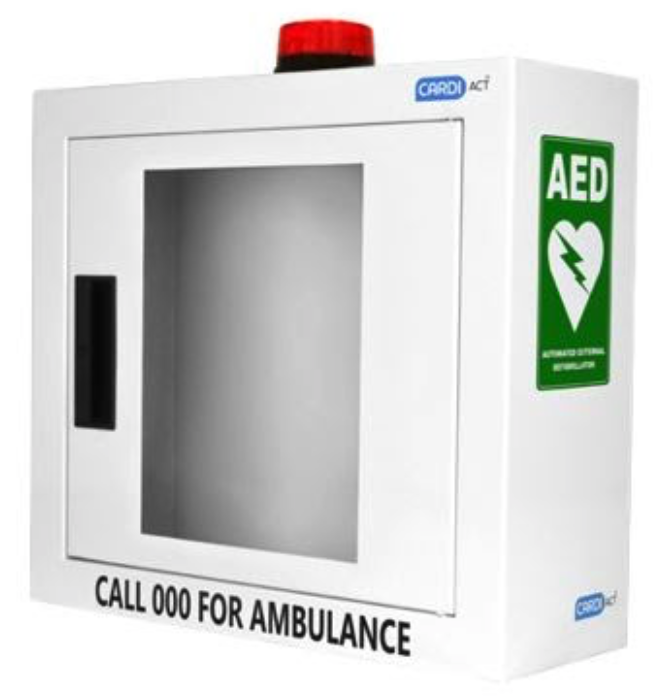


[0-10 numerical scale]

***How comfortable would you be using an AED in a situation of out of hospital cardiac arrest?***

Very comfortable

Slightly comfortable

Neutral

Slightly uncomfortable

Very uncomfortable

***How likely would you be to use an AED in a situation of out of hospital cardiac arrest?***

Very likely

Slightly likely

Neutral

Slightly unlikely

Very unlikely

I have only completed this survey once 🞏 Yes

Thank you for completing this questionnaire. Your answers will assist research into the use of signs and cabinets to identify automated external defibrillators (AEDs) in situations of out-of-hospital cardiac arrest.

AED Survey

**Signage of Automated External Defibrillators (AED) for Global Identification**

**STATISTICAL REPORT**

**Date: 7^th^ July 2022**

# Synopsis

A cross sectional survey was conducted to explore views on the ease to identify automated external defibrillator signage and cabinets in an emergency such as a cardiac arrest.

## Primary objective

To identify an appropriate means for identifying the AED signage to quickly respond to cardiac response events in the community.

## Trial Design

Cross sectional survey.

**Sample Size:**

The primary objective of this study is to estimate the proportion of the community that would find the yellow sign easier to identify in an emergency such as a cardiac arrest and the proportion of the community that would find the yellow cabinet easier to identify in an emergency such as a cardiac arrest. From pilot data it has been estimated that almost 90% of the community feel that the new AED cabinet stands out more than the current AED cabinet. It is therefore expected that the proportion of the community that would find the yellow cabinet easier to identify in an emergency such as a cardiac arrest would be quite high. This is similar for the sign. Table Y shows the accuracy of this estimate of the proportion for various sample sizes.

Table Y

| Proportion | 95% Confidence interval | Sample size |
| --- | --- | --- |
| 60% | (50.4, 69.6) | 100 |
|  | (55.7,64.3) | 500 |
|  | (56.0, 63.0) | 1000 |
| 70% | (61.0, 79.0) | 100 |
|  | (66.0, 74.0) | 500 |
|  | (67.2, 72.8) | 1000 |
| 80% | (72.2, 87.8) | 100 |
|  | (76.5, 83.5) | 500 |
|  | (77.5, 82.5) | 1000 |
| 90% | (84.1, 95.6) | 100 |
|  | (87.4, 92.6) | 500 |
|  | (88.1, 91.9) | 1000 |

Another important objective is to estimate the net promoter score (NPS) of the new AED cabinet and the current AED cabinet. The NPS is the proportion who would recommend the new cabinet to their friends and family (promoters) minus those that would not (demoters). In the pilot study, 88% would agree that this new AED cabinet is a better alternative so the NPS is expected to be quite high. The definition is shown below.

NPS= PR/T – D/T, where

PR=the number of promoters whose score is 9 or 10 for the question of whether they would recommend the new AED cabinet to a friend or family member,

D =the number of detractors whose score if between 0 and 6 inclusive, for the question of whether they would recommend the new AED cabinet to a friend or family member,

T = the total number of responders.

The variance of NPS is var(NPS)= (1-NPS)^2^xPR + (0-NPS)^2^xPA + (-1-NPS)^2^XD, where

PA= the number of promoters whose score is 7 or 8 for the question of whether they would recommend the new AED cabinet to a friend or family member,

So the standard error is 100 x var(NPS)/T

Table X: Various sample sizes and 95% confidence interval for various NPS

| **NPS** | **T= total number of responders** | Examples of combinations | | |  |
| --- | --- | --- | --- | --- | --- |
|  |  | Promotors | Detractors | Passive | 95% confidence interval |
| **95%** | 100 | 95% | 0% | 5% | (90.7, 99.3) |
|  | 500 |  |  |  | (93.1, 96.9) |
|  | 1000 |  |  |  | (93.6, 96.4) |
|  | 100 | 96% | 1% | 3% | (88.6, 99.4) |
|  | 500 |  |  |  | (91.6. 96.4) |
|  | 1000 |  |  |  | (92.3, 95.7) |
|  | 100 | 97% | 2% | 1% | (86.6, 99.4) |
|  | 500 |  |  |  | (90.2, 95.8) |
|  | 1000 |  |  |  | (91.0, 95.0) |
| **90%** | 100 | 95% | 5% | 0% | (81.5, 98.2) |
|  | 500 |  |  |  | (86.2, 93.8) |
|  | 1000 |  |  |  | (87.3, 92.7) |
|  | 100 | 93% | 3% | 4% | (82.4, 97.6) |
|  | 500 |  |  |  | (86.6, 93.4) |
|  | 1000 |  |  |  | (87.6, 92.4) |
|  | 100 | 92% | 2% | 6% | (82.9, 97.1) |
|  | 500 |  |  |  | (86.8, 93.2) |
|  | 1000 |  |  |  | (87.8, 92.2) |
| **85%** | 100 | 90% | 5% | 5% | (75.7, 94.3) |
|  | 500 |  |  |  | (80.8, 89.2 |
|  | 1000 |  |  |  | (82.0, 88.0) |
|  | 100 | 85% | 0% | 15% | (78.0, 92.0) |
|  | 500 |  |  |  | (81.9, 88.1) |
|  | 1000 |  |  |  | (82.8, 87.2) |
| **80%** | 100 | 85% | 5% | 10% | (70.0, 90.0) |
|  | 500 |  |  |  | (75.5, 84.5) |
|  | 1000 |  |  |  | (76.8, 83.2) |

For example, if the observed NPS is 80%, then a sample size of 500 would allow us to rule out a population NPS of 75% or lower.

**Data Analysis Plan**

Quantitative data will be analysed using descriptive statistics.

The proportion of the community that would find the new sign easier to identify in an emergency such as a cardiac arrest, and similarly for the cabinet, will be estimated with the 95% confidence interval. Logistic regression models will be used to assess the effect of age, ethnicity and region on this proportion.

NPS estimates will be calculated and 95% confidence intervals presented for the new and original sign and cabinet. Ordinal regression models will be used to assess the effect of age, ethnicity and region on the NPS is for each cabinet and sign. The McNemar-Bowker Test will be used to compare the distribution of promoters, passive and detractors between the new and original signs and similarly for the cabinets.

Participants will be able to view the study findings via the publication in a peer-reviewed medical journal.

# ANALYSIS

## Analysis Principles

P-values of less than 0.05 will be considered statistically significant unless stated otherwise.

## Definition of net promoter score (NPS)

NPS= PR/T – D/T, where

PR=the number of promoters whose score is 9 or 10 for the question of whether they would recommend the new AED cabinet to a friend or family member,

D =the number of detractors whose score if between 0 and 6 inclusive, for the question of whether they would recommend the new AED cabinet to a friend or family member,

T = the total number of responders.

The variance of NPS is var(NPS)= (1-NPS)^2^xPR + (0-NPS)^2^xPA + (-1-NPS)^2^XD, where

PA= the number of promoters whose score is 7 or 8 for the question of whether they would recommend the new AED cabinet to a friend or family member,

So the standard error is 100 x var(NPS)/T

# RESULTS

## Baseline Characteristics

The cohort demographics and preferences are shown in Table 1. Participants were allowed to check as many ethnicities as they wanted so they are not mutually exclusive.

***Table 1: Baseline Variables***

|  | *Baseline Variables* | |
| --- | --- | --- |
|  | | *Number (%)* |
| Total |  | 2538 |
| Gender | Female | 1454 (59.4%) |
|  | Male | 897 (36.6%) |
|  | Non-binary | 70 (2.9%) |
|  | Prefer not to say | 27 (1.1%) |
| Age | Mean (SD) | 30.9 (14.9) |
| Health Care Worker? | Yes | 510 (21.0%) |
|  | No | 1923 (79.0%) |
| White? | No | 483 (19.0%) |
|  | Yes | 2055 (81.0%) |
| African American? | No | 2514 (99.1%) |
|  | Yes | 24 (0.9%) |
| Asian? | No | 2245 (88.5%) |
|  | Yes | 293 (11.5%) |
| Hispanic? | No | 2504 (98.7%) |
|  | Yes | 34 (1.3%) |
| ATSI*? | No | 2452 (96.6%) |
|  | Yes | 86 (3.4%) |
| Pacific Islander? | No | 2502 (98.6%) |
|  | Yes | 36 (1.4%) |
| American Indian? | No | 2531 (99.7%) |
|  | Yes | 7 (0.3%) |

****Aboriginal and Torres Strait Islanders***

## Likelihood to recommend and preference results

Likelihood to recommend and preference results.

***Table 2: Preference outcomes***

| *Question* | | *Number (%)* |
| --- | --- | --- |
| Likelihood recommend Green AED SIGN | 0 (Not at all) | 113 (4.7%) |
|  | 1 | 23 (1.0%) |
|  | 2 | 85 (3.5%) |
|  | 3 | 110 (4.6%) |
|  | 4 | 157 (6.5%) |
|  | 5 (Neutral) | 742 (30.8%) |
|  | 6 | 220 (9.1%) |
|  | 7 | 238 (9.9%) |
|  | 8 | 256 (10.6%) |
|  | 9 | 137 (5.7%) |
|  | 10 (Extremely likely) | 331 (13.7%) |
| Likelihood recommend Yellow AED SIGN | 0 | 35 (1.4%) |
|  | 1 | 7 (0.3%) |
|  | 2 | 14 (0.6%) |
|  | 3 | 19 (0.8%) |
|  | 4 | 28 (1.2%) |
|  | 5 | 293 (12.1%) |
|  | 6 | 101 (4.2%) |
|  | 7 | 249 (10.3%) |
|  | 8 | 378 (15.7%) |
|  | 9 | 214 (8.9%) |
|  | 10 | 1077 (44.6%) |
| Likelihood recomm Green AED CABINET | 0 (Not at all) | 205 (8.5%) |
|  | 1 | 62 (2.6%) |
|  | 2 | 156 (6.4%) |
|  | 3 | 211 (8.7%) |
|  | 4 | 218 (9.0%) |
|  | 5 (Neutral) | 679 (28.0%) |
|  | 6 | 226 (9.3%) |
|  | 7 | 223 (9.2%) |
|  | 8 | 161 (6.6%) |
|  | 9 | 73 (3.0%) |
|  | 10 (Extremely likely) | 209 (8.6%) |
| Likelihood recomm Yellow AED CABINET | 0 (Not at all) | 30 (1.2%) |
|  | 1 | 7 (0.3%) |
|  | 2 | 15 (0.6%) |
|  | 3 | 15 (0.6%) |
|  | 4 | 26 (1.1%) |
|  | 5 (Neutral) | 166 (6.9%) |
|  | 6 | 87 (3.6%) |
|  | 7 | 195 (8.1%) |
|  | 8 | 369 (15.3%) |
|  | 9 | 293 (12.1%) |
|  | 10 (Extremely likely) | 1209 (50.1%) |
| How comfortable use an AED for OHCA? | Very comfortable | 631 (26.0%) |
|  | Slightly comfortable | 684 (28.2%) |
|  | Neutral | 344 (14.2%) |
|  | Slightly uncomfortable | 499 (20.5%) |
|  | Very uncomfortable | 271 (11.2%) |
| How likely use an AED for OHCA? | Very likely | 1013 (42.0%) |
|  | Slightly likely | 536 (22.2%) |
|  | Neutral | 415 (17.2%) |
|  | Slightly unlikely | 233 (9.7%) |
|  | Very unlikely | 217 (9.0%) |
| Sign preference | Yellow | 1778 (73.0%) |
|  | Green | 658 (27.0%) |
| Cabinet preference | Yellow | 2139 (87.6%) |
|  | White | 302 (12.4%) |

## Yellow Sign Preferences

The following shows the results of the proportion who find the **yellow sign** easier to identify in an emergency by demographic characteristics.

***Figure 1: Proportion who find the Yellow Sign easier to identify by demographic characteristics***
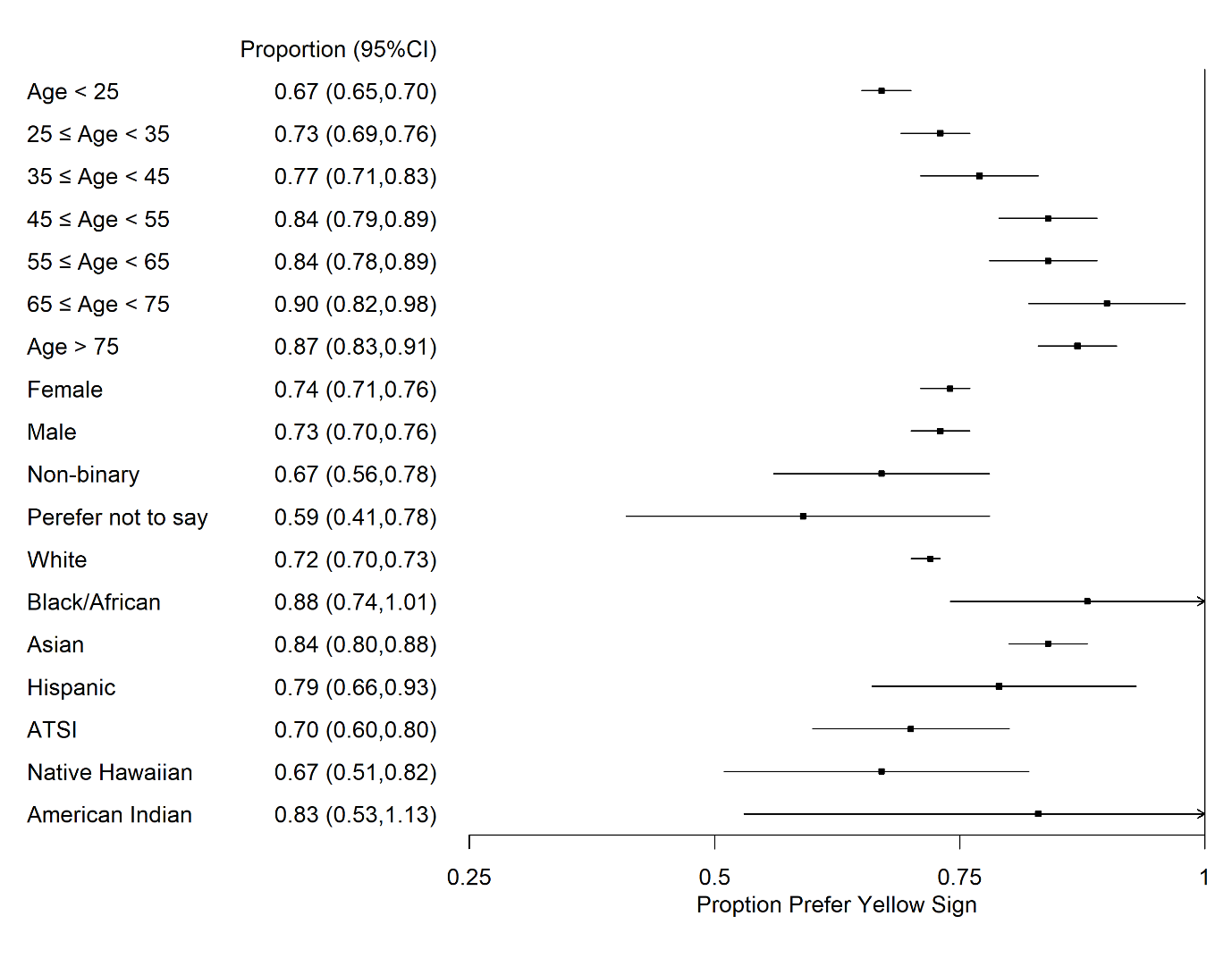


The following shows the results of the proportion who find the **yellow cabinet** easier to identify in an emergency by demographic characteristics.

***Figure 2: Proportion who find the Yellow Cabinet easier to identify by demographic***


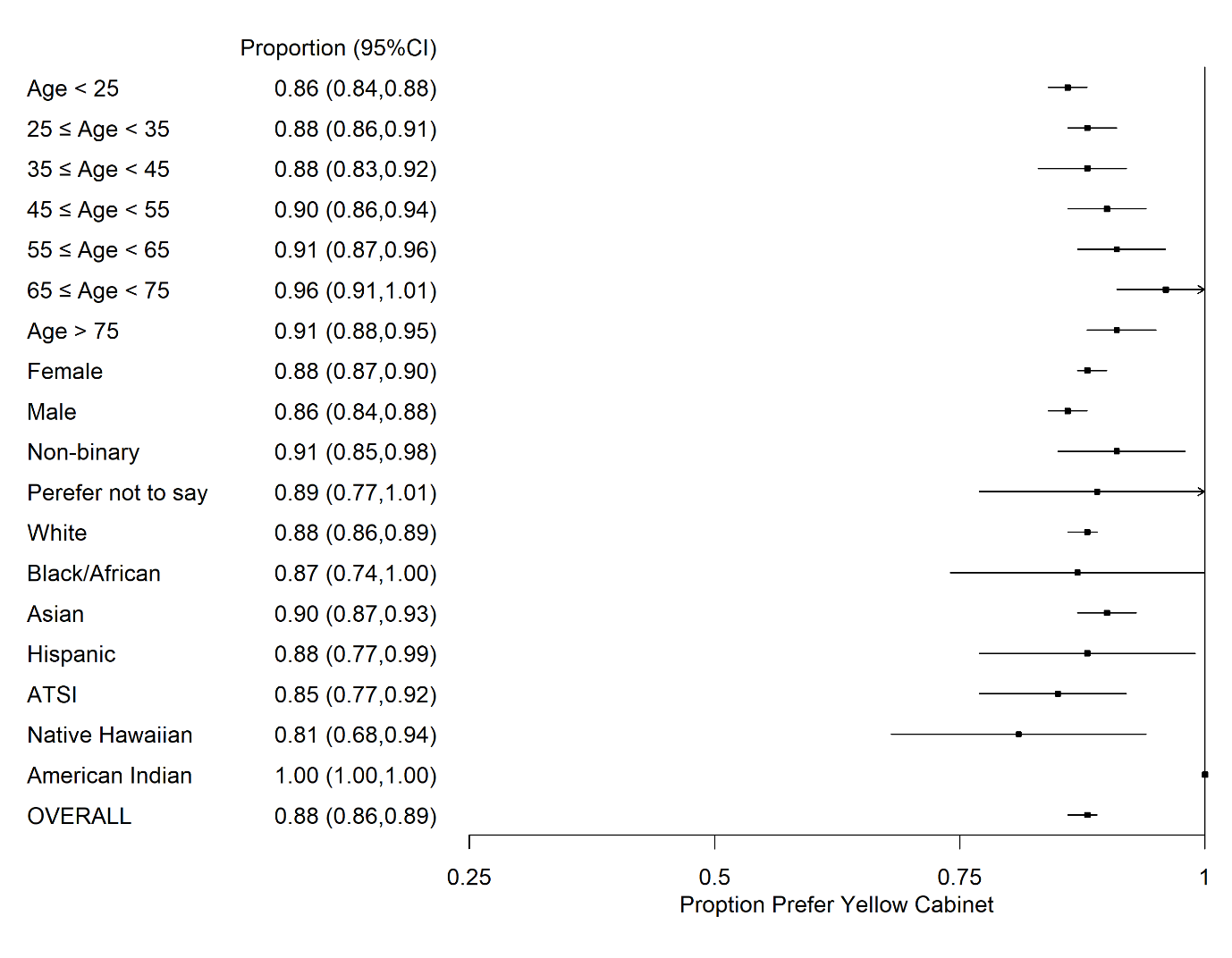


## Demographic variables associated with which is easier to identify

A logistic regression was used to look at the association with demographic variables and which sign and cabinet were more easy to identify in an emergency. For the sign, every year older the person was the odds of finding the yellow sign easier to identify increased by 2%. Ethnicities were not mutually exclusive, so they had to each go in the model as separate variables. The results are shown in Table 3.

***Table 3: Variables associated with stronger ease for identifying sign and cabinets for each colour***

|  | Odds Ratio and 95% confidence interval | p-value |
| --- | --- | --- |
| Variables found associated with easily identifying the **yellow sign compared to the green sign** |  |  |
| Age | 1.024 (1.016,1.031) | <0.0001 |
| Ethnicity: Asian | 1.86 (1.33,2.60) | 0.0003 |
| Variables found associated with easily identifying the **yellow cabinet compared to the green cabinet** |  |  |
| Age | 1.014 (1.004,1.023) | 0.0038 |
| Ethnicity: Asian | 1.70 (1.08, 2.77) | 0.0213 |
| Ethnicity: White | 1.89 (1.08, 3.33) | 0.0269 |

Showing the raw data for these significant variables in Table 4.

***Table 4: Key variables associated with stronger ease for identifying sign and cabinets for each colour***

|  | **Which is easier to identify in an emergency?** | |
| --- | --- | --- |
|  | **Yellow sign** | **Green sign** |
| **Age Mean (SD)**  **Median (IQR)** | 32.2 (15.6)  26 (20, 42) | 27.3 (12.1)  24 (19, 31) |
| **Asian Yes** | 84.2% (246/ 291) | 15.8% (46/291) |
| **No** | 71.5% (1533/2145) | 28.5% (612/2145) |
|  | **Yellow cabinet** | **White/Green cabinet** |
| **Age Mean (SD)**  **Median (IQR)** | 31.3 (15.1)  26 (20, 40) | 28.4 (13.6)  24 (19, 33) |
| **Asian Yes** | 90.0% (262/291) | 10.0% (29/291) |
| **No** | 87.3% (1877/2150) | 12.7% (273/2150) |
| **White Yes** | 87.7% (1797/2048) | 12.3% (251/2048) |
| **No** | 87.0% (342/393) | 13.0% (51/393) |

## Net Promoter Score

The net promoter score is shown in the table below. Net Promoter Score (NPS) is a customer loyalty and satisfaction measurement taken from asking customers how likely they are to recommend your product or service to others on a scale of 0-10.

***Table 5: Net Promoter Score calculations***

|  | Promoters | Detractors | Passive | Total | NPS | Variance | Stand Error |
| --- | --- | --- | --- | --- | --- | --- | --- |
| Green-white sign | 137+331  =468 | 113+23+85  +110+157  +742+220  =1450 | 238+256  =494 | 1450+494  +468  =2412 | (468-1450)/2412 =-0.407 | (1+ 0.407)^2^x468/2412+ (0 + 0.407)^2^x494/2412 + (-1+0.407)^2^ x 1450/2412 = 0.6294351 | (0.6294351/2412)  =0.01615425 |
| Yellow-red sign | 1077+214  =1291 | 35+7+14+19+28+293+101  =497 | 249+378  =627 | 1291+497  +627  =2415 | (1291-497)/2415  =0.331 | (1- 0.331)^2^x1291/2415+ (0 -0.331)^2^x627/2415 + (-1-0.331)^2^ x 497/2415 = 0.6322823 | (0.6322823/2415)  =0.01618069 |
| Green-white cabinet | 209+73  =282 | 205+62+156+211+218+679+226  =1757 | 223+161  =384 | 282+1757  +384  =2423 | (282-1757)/2423  =-0.609 | (1+ 0.609)^2^x282/2423+ (0 + 0.609)^2^x384 /2423+ (-1+0.609)^2^ x 1757 /2423= 0.4709429 | (0.4709429/2423)  = 0.01394143 |
| Yellow-red cabinet | 1209+293  =1503 | 30+7+15+15+26+166+87  =346 | 195+369  =564 | 1503+346  +564  =2413 | (1502-346)/2413  =0.479 | (1- 0.479)^2^x1503/2413+ (0 - 0.479)^2^x564/2413 + (-1-0.479)^2^ x 346/2413 = 0.5363594 | (0.5363594/2413)  = 0.01490903 |

Where PR=the number of promoters whose score is 9 or 10 for the question of whether they would recommend the new AED cabinet to a friend or family member,

D =the number of detractors whose score if between 0 and 6 inclusive, for the question of whether they would recommend the new AED cabinet to a friend or family member,

T = the total number of responders.

The variance of NPS is var(NPS)= (1-NPS)^2^xPR/T + (0-NPS)^2^xPA/T + (-1-NPS)^2^XD/T, where

PA= the number of promoters whose score is 7 or 8 for the question of whether they would recommend the new AED cabinet to a friend or family member,

The net promoter score is shown in the table below.

***Table 6: Net Promoter Score results***

|  | **Promoters** | **Detractors** | **Passive** | **NPS and 95% CI** |
| --- | --- | --- | --- | --- |
| **Green-white sign** | 19.4% | 60.1% | 20.5% | -0.41, (-0.44, -0.38) |
| **Yellow-red sign** | 53.5% | 20.6% | 26.0% | 0.33 (0.30,0.36) |
| **Green-white cabinet** | 11.6% | 72.5% | 15.8% | -0.61 (-0.64, -0.58) |
| **Yellow-red cabinet** | 62.3% | 14.3% | 23.4% | 0.48 (0.45, 0.51) |
